# Multiple roles of mucins in pancreatic cancer, a lethal and challenging malignancy

**DOI:** 10.1038/sj.bjc.6602163

**Published:** 2004-10-19

**Authors:** N Moniaux, M Andrianifahanana, R E Brand, S K Batra

**Affiliations:** 1 Department of Biochemistry and Molecular Biology, Eppley Institute for Research in Cancer and Allied Diseases, University of Nebraska Medical Center, Omaha NE 68198, USA; 2 Evanston Northwestern Healthcare, Evanston, IL 60201, USA

**Keywords:** mucin, MUC1, MUC4, pancreatic cancer, diagnosis, therapy

## Abstract

Mucins are members of an expanding family of large multifunctional glycoproteins. Pancreatic mucins have important biological functions, including the protection, lubrication, and moisturisation of the surfaces of epithelial tissues lining ductal structures within the pancreas. Several lines of evidence support the notion that deregulated mucin production is a hallmark of inflammatory and neoplastic disorders of the pancreas. Herein, we discuss the factors that contribute to the lethality of pancreatic cancer as well as the key role played by mucins, particularly MUC1 and MUC4, in the development and progression of the disease. Aspects pertaining to the aberrant expression and glycosylation of mucins are discussed, with special emphasis on their potential impact on the design and implementation of adequate diagnostic and therapeutic strategies for combating this lethal malignancy.

Mucins are a large family of glycoproteins that, either directly or indirectly, act to maintain the integrity, lubricate and protect the epithelial surfaces within the human body. In general, mucins exhibit a defined spatial and temporal pattern of expression throughout the development of an organism (Braga *et al*, 1992). In numerous pancreatic pathologic situations, however, their expression is deregulated, and their aberrant expression is often associated with a poor prognosis (Braga *et al*, 1992; Reis *et al*, 1999; Andrianifahanana *et al*, 2001). This paper will review the role played by mucins, particularly MUC1 and MUC4, in the development and progression of pancreatic cancer (PC) and will highlight key aspects that may account for the lethality of this malignancy.

## THE MUCIN FAMILY

For long, mucins were known as high molecular weight *O*-glycoproteins, secreted by specialised epithelial cells. As main contributors to the rheologic properties of the mucus, mucins were thought to have the sole functions of protecting and lubricating epithelial surfaces. Following the development of molecular biological methods, however, a wide range of mucin structures became available and gave rise to a plethora of biochemical definitions of mucins. As the diversity of mucin structures grew in importance, a variety of functions were assigned accordingly. At present, a total of 21 genes have received the appellation MUC: *MUC1-2*, *MUC3A*, *MUC3B*, *MUC4*, *MUC5AC*, *MUC5B*, *MUC6-13*, *MUC15-20* (Moniaux *et al*, 2001; Hollingsworth and Swanson, 2004; Andrianifahanana *et al*, 2004). In all, 14 of these code for proteins presenting the five common features of a mucin, including (I) secretion into the mucus layer, (II) high molecular weight *O*-glycoprotein, (III) presence of a tandem repeat array encoded by a unique and centrally positioned large exon, (IV) presence of a predicted peptide domain containing a high percentage of serine and threonine residues, and (V) a complex pattern of mRNA expression.

The 14 classical mucins can be further grouped into two subfamilies: secreted and membrane-bound. Typically, secreted mucins are expressed exclusively by specialised epithelial cells, are secreted in the mucus, and display a restricted expression pattern within the human body. Among these, MUC2, MUC5AC, MUC5B, and MUC6 are expressed in the pancreas either under normal physiologic or tumoral conditions. These four mucins, referred to as the gel-forming mucins, have a common architecture with a high level of similarity to the pro-von Willebrand factor. The secreted mucins whose genomic sequences have been fully characterised are known to harbour five D domains, thus called because of their homology to the D domains of the von Willebrand factor. The D1, D2, D′, and D3 domains are located in the N-terminal region and the D4 and CK (Cystine Knot) domains in the C-terminal region. Moreover, cystein-rich domains (called Cys) alternate with the tandem repeat sequences in a variable number depending on the mucin (for a review, see Moniaux *et al*, 2001). The gel-forming mucins form intermolecular disulphide-linked multimers. Mucin subunits initially form homodimers through the disulphide bonds from their CK domains and subsequently hetero-oligomerise through the D domains from their N-terminal extremities (Van Klinken *et al*, 1998). The Cys domains are believed to bring another level of complexity to the oligomerisation process via intermolecular disulphide bond formation. As the composition of gel-forming mucins differs among tissues according to the types of mucins expressed, the rheologic properties of the mucus will be different and specific.

The membrane-bound mucins are composed of MUC1, MUC3A, MUC3B, MUC4, MUC11, MUC12, MUC16, and MUC17. For the purpose of clarity, MUC11 and MUC12 will be hereon referred to as MUC11-12, and MUC3A and MUC3B as MUC3A-B. It is not fully understood at this point whether these genes are related to unique or independent loci. The membrane-bound mucins share a common property of being expressed by distinct cellular types, epithelial or other. Implanted at the apical surface of epithelial cells, they are also secreted and, therefore, take part in mucus formation. As compared to the secreted mucins, they present a wider and more complex expression pattern. Indeed, they can be expressed in four distinct forms: membrane-anchored, soluble (proteolytic cleavage of the membrane-bound form), secreted (alternatively spliced variants), and lacking the tandem repeat array (alternatively spliced variants) (Wreschner *et al*, 1990; Choudhury *et al*, 2000; Moniaux *et al*, 2000). The ratio of one form to another appears to be tissue-specific and associated to the physiologic conditions (normal or tumoral). In addition to the proteolytic cleavage that releases the secreted forms from the cell surface, mucin precursors from this group, MUC1 and MUC4, possess a second proteolytic cleavage site that processes the precursor into a mature heterodimer. This second cleavage is thought to confer upon mucins their functional conformation. Membrane-anchored mucins contain a SEA (Sea urchin sperm protein, Enterokinase and Agrin) module, with the exception of MUC4. MUC3A-B, MUC4, MUC11-12, and MUC17 contain two to three epidermal growth factor (EGF)-like domains.

**Figure 1:**
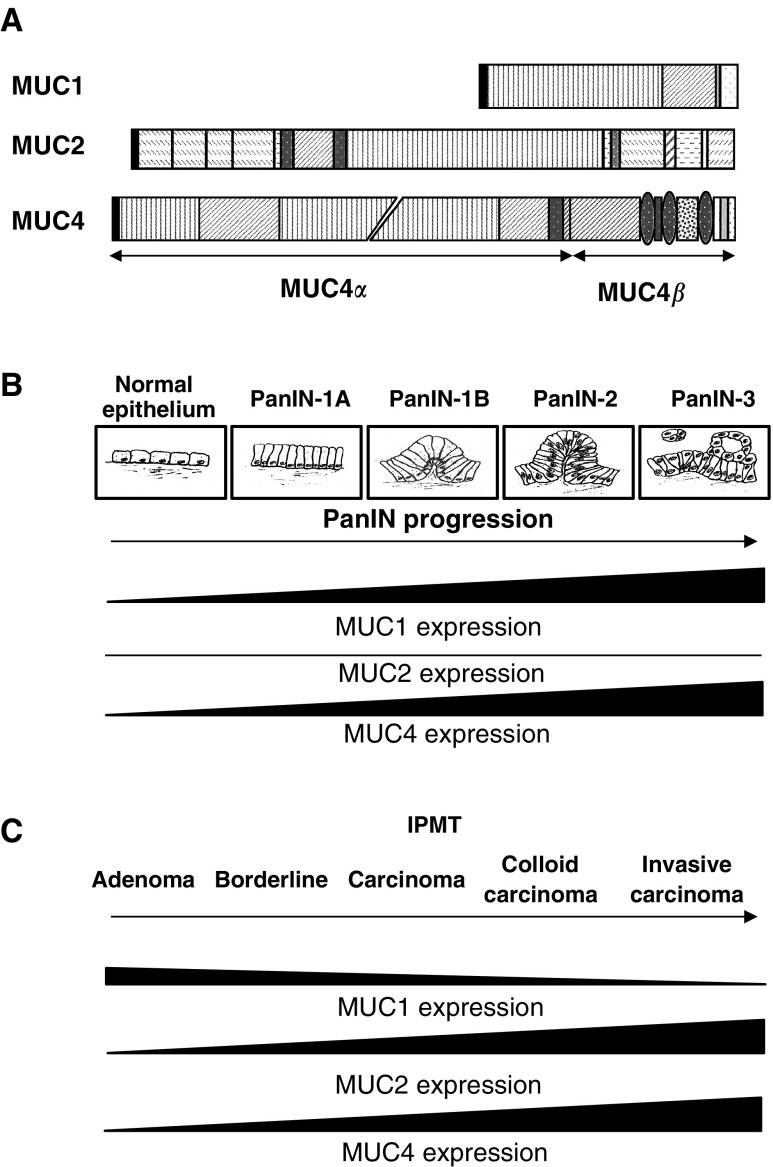
(**A**) Schematic representation of the deduced amino-acid sequence of the MUC1, MUC2, and MUC4 genes, which are deregulated in PC. The MUC1 apomucin has an apparent simple structure with a central domain composed of sequences repeated in tandem, a transmembrane sequence, and a cytoplasmic tail. MUC4 is a heterodimeric protein, composed of an 850-kDa mucin-type *α* subunit, noncovalently linked to an 80-kDa growth-factor-like membrane-bound *β* subunit. MUC2 is a secreted mucin with several domains similar to the von Willebrand factor. Keys to figure: black, signal peptide; vertical lines, tandem repeat; diagonal lines serine, threonine-rich nonrepetitive sequence domain; dense dots, domain rich in N-glycosylation sites; open box, unique sequence; hatched boxes, cystein-rich domain; hatched ovals, EGF-like domain; light grey, transmembrane domain; small dots, cytoplasmic tail; descending hatched lines, D domains; ascending hatched lines, CK domain; horizontal lines, C domain; dense vertical lines, B domain. (**B**) Schematic representation of the PanIN lesions and MUC1, 2, and 4 expression during the progression of PC. Pancreatic intraepithelial neoplasias are thought to mimic the development of PC, evolving from PanIN-1A (flat epithelial lesions composed of tall columnar cells with basally located nuclei) to PanIN-3 (papillary lesion with budding off of small clusters of epithelial cells into the lumen). (**C**) Expression of MUC1, MUC2, and MUC4 in IPMT.

Among the membrane-bound mucins, MUC1 and MUC4 are the two main mucins associated with PC. Although they both belong to the same mucin subfamily, MUC1 and MUC4 present significantly distinct structures. MUC1 has an apparent simple architecture. The *MUC1* gene (Figure 1A

) codes for a membrane-associated protein with distinct domains: an amino-terminal domain consisting of a 20-amino-acid signal peptide and degenerate tandem repeats; a large central domain (two-thirds of the core protein sequence) made up of variable numbers of a 20-amino acid repeat; and a carboxyl terminus made of degenerate tandem repeats, a hydrophobic membrane-spanning domain of 31 amino acids, and a cytoplasmic domain of 69 amino acids. In normal individuals, there is a variation in the number of repeats per allele (from 25 to >125 repeats), implying that the length of the repetitive array may not be critical for the normal function of MUC1. MUC4, on the other hand, is one of the largest human membrane-anchored mucins identified to date (Figure 1A). The amino-terminus of *MUC4* is comprised of a 27-residue signal peptide, followed by three imperfect repetitions of a motif, varying from 126 to 130 residues and by a unique sequence of 554 residues. The central and largest domain is comprised of a perfect tandem repetition of a 16-amino-acid residue motif that could be repeated up to 400 times. Over 20 distinct alleles have been identified for MUC4. These show a variation in the size (6.5–19 kb) of the tandem repeat array. It is not as yet clear whether the size of the tandem repeat is important for the functions or expression of MUC4. The C-terminal region can be divided into 12 domains (CT1 to CT12). CT6, CT7, and CT9 are EGF-like domains, and CT11 corresponds to the transmembrane domain. A putative proteolytic cleavage site (a GDPH motif) is found between domains CT4 and CT5. A MUC4 precursor of 930 kDa yields at least two subunits (MUC4*α* and MUC4*β*) following proteolytic cleavage. Hence, MUC4 is predicted to be a membrane-associated mucin that extends up to 2.12 *μ*m above the cell surface. MUC4*α* is an extracellular mucin-type glycoprotein subunit, whereas MUC4*β* is a growth factor-like transmembrane subunit (Moniaux *et al*, 1999). Both the *MUC1* and *MUC4* genes encode numerous alternatively splice forms, some of which are variants devoid of the mucin hallmark, the tandem repeat array. These splice forms, called MUC1/Y, MUC1/X, MUC1/Z, MUC4/X, and MUC4/Y, present a growth factor-like architecture and are thought to be involved in fetal and tumour development. However, the lack of specific antibodies makes it difficult to perform appropriate functional studies.

## ABNORMAL EXPRESSION OF MUCIN GENES IN PC

Of particular interest, the pattern of mucin expression within the pancreas varies depending on the pathophysiologic conditions (Table 1

**Table 1:** Mucin gene expression in different pancreatic physiologic conditions

						**Adenocarcinoma**	
**Gene**	**Fetus**	**Normal pancreas**	**Pancreatitis**	**IPMT**	**PanIN**	**Tissue**	**Cell line**
MUC1	−/+	+	++	+	++	+++	+++
MUC2	−	−	−	+++	−	−	−/++
MUC3	−	±	±	±	ND	±	−/+
MUC4	−	−	−	++	+ to +++	+++	+++
MUC5AC	−	+	−/+	++	ND	−/+	−/+
MUC5B	−	+	++	++	ND	+/++	−/++
MUC6	+	++	++/+++	++	ND	++/+++	−/+++
MUC11/12	ND	−/+	ND	ND	ND	ND	ND
MUC16	ND	ND	ND	ND	ND	++	ND
MUC17	ND	ND	ND	ND	ND	ND	+

−=negative; +=low; ++=moderate; +++=intense; ND=not determined; −/+=from negative to positive depending the sample; ±=negative or positive depending the reports; IPMT=intraductal papillary-mucinous tumours; PanIN=pancreatic intraepithelial neoplasias.

). The main mucin expressed by the normal pancreatic tissue is MUC6 (Kim *et al*, 2002). MUC6 and MUC1 are already expressed at an early stage of gestation in the fetal pancreas (Reid and Harris, 1998; Buisine *et al*, 2000; Adsay *et al*, 2002). MUC5B is moderately expressed, while MUC1 and MUC5AC are weakly detected in the adult pancreas (Yonezawa *et al*, 1997; Yonezawa *et al*, 1999; Andrianifahanana *et al*, 2001; Terris *et al*, 2002). We have previously reported that the overexpression of the *MUC1* gene as well as the aberrant expression of *MUC4* were associated with the development and progression of PC (Swartz *et al*, 2002; Park *et al*, 2003). In the normal pancreas, the expression of MUC1 is confined to the apical membrane of the intralobular ductules. It is neither expressed in the larger ducts nor in the islet of langerhans. The overexpression of MUC1 is observed during the early stages of the development of PC, which is further increased in invasive carcinoma (Levi *et al*, 2004). On the other hand, MUC4 is minimally or not expressed in the normal pancreas or chronic pancreatitis but is highly expressed in human pancreatic tumours and pancreatic tumour cell lines (Balague *et al*, 1994; Choudhury *et al*, 2000; Andrianifahanana *et al*, 2001).

Lesions known as pancreatic intraepithelial neoplasia (PanIN) are thought to represent the early stages of PC development (Figure 1B). They progress from flat to papillary without atypia, to papillary with atypia, and to lesions with severe atypia and are classified PanIN-1A to PanIN-1B, PanIN-2, and PanIN-3 (Hruban *et al*, 2001). Atypical intraductal lesions can progress to invasive adenocarcinoma (Brat *et al*, 1998). MUC4 is expressed by metasplastic ducts and its expression increases with a higher grade of PanINs (Swartz *et al*, 2002; Park *et al*, 2003). Similarly, MUC1 expression also increases with the progression of PanIN lesions (Figure 1B), whereas MUC2 expression remains unchanged (Adsay *et al*, 2002).

*MUC* gene expression has not been extensively studied in intraductal papillary-mucinous tumours (IPMTs) of the pancreas. MUC2 and MUC5AC levels were shown to be very high in IPMT compared to adenocarcinomas (Terada and Nakanuma, 1996; Yonezawa *et al*, 1997). An altered expression of *MUC* genes (overexpression of MUC2 and MUC5AC) was seen in IPMTs, which could be correlated with types and lesions (Figure 1C) (Terris *et al*, 2002). The main difference between adenocarcinoma of the pancreas and IPMT is an increased expression of MUC2 associated with a decreased expression of *MUC1* in IPMT *vs* adenocarcinoma. (Adsay *et al*, 2002) In both cases, MUC4 is highly aberrantly expressed. While mucin gene profile is in general a good marker for PC, it also serves to distinguish IPMT from adenocarcinoma.

Other mucin genes, such as *MUC11* (Williams *et al*, 1999), *MUC12* (Williams *et al*, 1999), and *MUC17* (Gum, Jr *et al*, 2002) are detected in pancreatic tissues. However, their specific expression pattern in relation to PC development is still unclear.

## MUCINS FOR PC DIAGNOSTIC

The pancreas is the second largest gland of the digestive system following the liver. It lies retroperitoneally in the upper abdominal cavity, with its head enclosed by the curve of the duodenum and its tail reaching to the spleen. It performs both endocrine and exocrine functions. The exocrine component, constituting about 95% of the mature pancreas, is made up of 85% acinar cells and 10% of secreting ductal cells. The endocrine cells are believed to occupy just 1–2% of the total pancreatic volume in adults.

Adenocarcinomas of the ductal phenotype constitute over 85% of PC, and other tumours of ductal type, such as mucinous cystic tumours, serous cystic tumours and IPMTs make up another 5–7%. A number of factors, including hereditary, environmental, occupation, and social factors are now recognised as potential contributors to the development of PC (Michaud *et al*, 2003). Pancreatic cancer is an international problem because of its increasing incidence worldwide. The incidence and age-adjusted mortality rates are almost equal, underscoring the aggressive nature of the disease. It is a daunting challenge for modern medicine as the survival rates for patients diagnosed with metastatic PC range from 4 to 6 months on average. The lethality of the disease is best illustrated by its poor 5-year survival rate of a mere 5%. The highest cure rate only occurs if the tumour is truly localised to the pancreas; however, this stage of disease accounts for fewer than 20% of all cases. For those patients with localised disease and small tumour size (<2 cm) with no lymph node metastases and no extension beyond the ‘capsule’ of the pancreas, complete surgical resection can yield actuarial 5-year survival rates of 18–24% (Yeo *et al*, 1997, 1999). Unfortunately, the signs of early stage PC are vague and often attributed to other problems by both patients and physicians. More specific symptoms tend to develop after the tumour has grown to invade other organs or blocked the bile ducts. Patients are usually diagnosed at an advanced stage, with a high incidence of associated metastases, spread all over the body. The inability to diagnose PC at an early, localized, and curable stage has contributed to poor prognosis. The silent course of PC and its explosive fatal outcome have hindered studies of identification of early biochemical and genetic alterations that could help us diagnose the disease at a curable stage and develop successful therapeutic strategies. Thus, PC remains a dismal disease and early diagnostic markers and therapeutic targets are urgently needed.

Early symptoms of PC are difficult to detect and are often ignored. There are no tumour-specific markers for PC; markers such as serum CA 19-9, DUPAN-2, and CA125 that are being used as potential targets have low specificity (McLaughlin *et al*, 1999; Rhodes, 1999). For over two decades, the structures of these antigens (CA 19-9, DUPAN2, or CA 125) have been heavily investigated for the development of serum-based immunoassays to detect early cancers. These antigens contain oligosaccharide structures that are being recognised by monoclonal antibodies. Further, these saccharide epitopes are present on the *O*-glycosidic chains harboured by the apomucin backbones. For examples, MUC1 carries CA19-9 (Burdick *et al*, 1997) and DUPAN-2 (Khorrami *et al*, 2002) epitopes, and MUC16 carries CA 125 epitope (Yin *et al*, 2002). At this time, CA19-9 remains the only Food and Drug Administration (FDA)-approved marker to monitor PC. The CA 19-9 recognises a mucin-type glycoprotein sialosyl lewis a/b antigen. Since 5% of the general population are lewis a/b negative, the maximum sensitivity of this marker is of 95%.

Aberrant glycosylation occurs in essentially all types of experimental and human cancers and has been associated with tumorigenecity and metastasis. In fact, many glycosyl epitopes constitute tumour-associated antigens (TAAs) and have been used as a target for immunotherapy and diagnosis. The nature of aberrant glycosylation as a result or a cause of cancer remains enigmatic. Many recent studies have indicated that some, if not all, types of aberrant glycosylation are a result of initial oncogenic transformation, as well as a key event in induction of invasion and metastasis.

The mucin-type *O*-glycans are attached to the glycoproteins through *O*-glycosidic linkages between *N*-acetylgalactosamine and serine or threonine amino-acid residues in the apomucin moieties. *O*-glycans are assembled by a series of reactions catalysed by glycosyltransferases and sulphotransferases in the Golgi compartment. In cancer cells, the expression pattern of these enzymes is dysregulated and leads to tumour-specific glycanic epitopes. Depending on the glycosyltransferases expressed, several hundred different *O*-glycan structures have been described and classified according to their core structures. All of the cores have the *N*-acetylgalactosamine linked to the serine or threonine residues that represents the Tn antigen in common. When sialylated, this antigen forms the sialyl-Tn (sTn) epitope reported to be expressed on the surface of mucin cores. Indeed, sTn is detected at the surface of MUC1 overexpressed by pancreatic adenocarcinoma cells (Burdick *et al*, 1997). It has been proposed that the appearance of the sTn antigen might result from MUC1 overexpression. The abundance of acceptor sites may saturate the post-translational modification (Burdick *et al*, 1997). The sTn antigen is recognised by the mouse monoclonal antibody CC49, which is being extensively investigated for the development of radioimmunotherapeutic protocols.

Alternatively, the Tn antigen can be elongated to form one of the eight distinct cores and terminated with sulphate, sialic acid, fucose, galactose, *N*-acetylglucosamine, and *N*-acetylgalactosamine. Depending on the physiologic conditions, the peripheral sugar may vary and gives rise to tumor-associated antigens, such as Le^a^ (Lewis^a^), Le^b^, Le^x^, Le^y^, sLe^a^, sLe^c^, and sLe^x^. The CA 19-9 described previously recognises the sLe^a^ and DUPAN-2 recognises the sLe^c^.

## IMPLICATION OF MUCINS IN THE PATHOGENESIS OF PC

As described previously, both the glycosylation and the expression of mucins are deregulated during the development and progression of pancreatic adenocarcinoma. The main mucins that present the most deregulated expression patterns are MUC1 and MUC4, two mucins that belong to the membrane-anchored mucin subfamily. Therefore, this review will focus on the known functions of MUC1 and MUC4. Both MUC1 and MUC4 play a key role in the development of PC as well as in its progression (invasion and metastasis). As indicated earlier in this paper, the high incidence of metastases in PC is the major cause of its lethality. MUC1 and MUC4 are implicated in almost all of the steps associated with the development of metastases.

MUC1 and MUC4 possess antiadhesive properties. The negative charge of the *O*-glycosidic chains carried by their central repetitive domain provides a extended conformation to MUC1 and MUC4, with a size up to 500 nm and 2.12 *μ*m (Moniaux *et al*, 1999), respectively. Moreover, MUC1 and MUC4 are not only upregulated in PC but their upregulation is also accompanied by the loss of their strictly apical localisation. The steric hindrance caused by the overexpression of these two extended glycoproteins disturbs the cell–cell and/or cell–matrix interactions (Komatsu *et al*, 1997). This physical mechanism favours the release of cancer cells into the circulation. MUC1 also interacts directly with *β*-catenin via an SXXXXXSSL motif (where X represents any amino-acid residues) in its cytoplasmic tail (Yamamoto *et al*, 1997). *β*-Catenin plays important functions in the formation of the cell–cell junction via E-cadherin interaction (Hulsken *et al*, 1994). *β*-Catenin also binds to adenomatous polyposis coli (APC) (partner in the Wingless/Wnt-1 signal pathway). The Wnt-1 intracellular pathway is directly implicated in the development of the central nervous system. Complex binding to *β*-catenin is a mutually exclusive process. APC overexpression reduces the level of free cytoplasmic *β*-catenin, and thus reduces the formation of *β*-catenin/E-cadherin complexes and also decreases intercellular adherence. The formation of these complexes is regulated by the phosphorylation of the cytoplamic tail of each partner by GSK3*β*. After phosphorylation, *β*-catenin is degraded. GSK3*β* is also able to phosphorylate the *β*-catenin-binding site on the MUC1 cytoplasmic tail (Li *et al*, 1998). The phosphorylation at the serine residue of the cytoplasmic tail of MUC1 by GSK3*β* was shown to decrease the binding of MUC1 to *β*-catenin (Li *et al*, 1998). The relative level of MUC1, E-cadherin, *β*-catenin, GSK3*β*, and APC seem to be critical for maintaining epithelium integrity. The overexpression of MUC1 leads to an increase in the interaction between MUC1 and *β*-catenin, thereby inhibiting *β*-catenin/E-cadherin interaction and disrupting cell–cell contacts, which facilitate the release of the tumour cell from the tissue. The cytoplasmic tail of MUC1 is also phosphorylated at tyrosine residue. *In vitro*, tyrosine phosphorylation of the MUC1 cytoplasmic domain increases recolonisation and promotes changes in cell–cell adhesion (Quin and McGuckin, 2000). Interestingly, both src and EGFR induce tyrosine phosphorylation and enhance interactions between MUC1 and *β*-catenin (Li *et al*, 2001; Schroeder *et al*, 2001).

The expression of mucin-associated carbohydrate antigens, sLe^x^ and sTn, is associated with higher-grade PanIN lesions (Park *et al*, 2003) and is thought to result from a shift in the expression of glycosyltransferases. These mucin-associated antigens are known to bind endothelial cells through the P- and E-selectins, and the cell-adhesion molecule, ICAM-1 (Regimbald *et al*, 1996). The P-selectin ligand (PSGL-1) (Sako *et al*, 1993) presents a structure similar to MUC1 and MUC4 and carries within its repetitive domain *O*-glycosidic chains with terminal sugars comparable to those of MUC1 and MUC4 in tumoral situations. Therefore, in addition to favouring the release of PC cells, MUC1 and MUC4 also facilitate their extravasation into and/or from the circulation (Burdick *et al*, 1997). MUC4 is known to inhibit tumour killing by lymphokine-activated killer cells (Komatsu *et al*, 1999).

Information regarding the role of MUC1 and MUC4 in the growth of primary pancreatic tumours has remained scarce until recently. Both mucins have been reported to serve as potential modulators of the receptor tyrosine kinase signalling pathways. Indeed, both MUC1 and MUC4 have been referred to as the heterodimeric partners of EGFR/HER1 (Li *et al*, 2001; Schroeder *et al*, 2001) and HER2 (Carraway *et al*, 2002), respectively. When MUC1 interacts with EGFR, the cytoplasmic tail of MUC1 becomes phosphorylated and leads to the activation of c-Src (Li *et al*, 2001). Moreover, the formation of the complex activates the mitogenic MAP kinase pathway (Schroeder *et al*, 2001). For MUC4, its membrane-bound subunit acts as a ligand for HER2, activating this growth factor receptor, and in synergy with neuregulin, potentiates the phopshorylation of HER2 and HER3. Phosphorylation of HER2 occurs at residue Tyr1248 after binding with MUC4 and leads to the upregulation of the cell-cycle inhibitor, p27^kip^ (Jepson *et al*, 2002). However, in presence of neuregulin, MUC4/HER2 activation downregulates p27^kip^ but activates the protein kinase B/Akt via HER3. Therefore, MUC4 appears to be a modulator of cell proliferation. Inhibition of MUC4 expression using antisense or short-interfering RNA (siRNA) oligonucleotides specific to MUC4 results in a decreased tumorigenicity and dissemination of cancer cells (Singh *et al*, 2004). We believe that MUC4 is implicated in tumour growth and metastasis by directly altering the tumour cell properties (adhesion/aggregation and motility), and/or in part, via modulating HER2 expression.

## CONCLUSIONS AND PERSPECTIVES

Pancreatic cancer is an international problem because of its increasing incidence worldwide. The incidence and age-adjusted mortality rates are almost equal, underscoring the aggressive nature of the disease. Although efforts are being made to unveil the principles governing the initiation and progression of this cancer, and to identify the factors that confer its particular aggressiveness, the exact succession of molecular events underlying the development of this devastating malignancy has remained unsolved. The management of PC is, therefore, an ongoing challenge. Mucins have been implicated in the pathogenesis and progression of PC. Differential glycosylation, aberrant expression, and alternative splicing of mucins are being investigated in PC. MUC1 and MUC4 are finding unique functions in the early diagnosis and progression of the disease. The potential use of these mucins in the targeted therapy of pancreatic and other cancers is under active investigation.
